# Amniotic fluid stem cell extracellular vesicles as a novel fetal therapy for pulmonary hypoplasia: a review on mechanisms and translational potential

**DOI:** 10.1093/stcltm/szae095

**Published:** 2025-01-17

**Authors:** Fabian Doktor, Lina Antounians, Rebeca Lopes Figueira, Kasra Khalaj, Miriam Duci, Augusto Zani

**Affiliations:** Developmental and Stem Cell Biology Program, Peter Gilgan Centre for Research and Learning, The Hospital for Sick Children, Toronto, ON, Canada M5G 0A4; Division of General and Thoracic Surgery, The Hospital for Sick Children, Toronto, ON, Canada M5G 1X8; Department of Pediatric Surgery, Leipzig University, Leipzig 04109, Germany; Developmental and Stem Cell Biology Program, Peter Gilgan Centre for Research and Learning, The Hospital for Sick Children, Toronto, ON, Canada M5G 0A4; Division of General and Thoracic Surgery, The Hospital for Sick Children, Toronto, ON, Canada M5G 1X8; Developmental and Stem Cell Biology Program, Peter Gilgan Centre for Research and Learning, The Hospital for Sick Children, Toronto, ON, Canada M5G 0A4; Division of General and Thoracic Surgery, The Hospital for Sick Children, Toronto, ON, Canada M5G 1X8; Developmental and Stem Cell Biology Program, Peter Gilgan Centre for Research and Learning, The Hospital for Sick Children, Toronto, ON, Canada M5G 0A4; Division of General and Thoracic Surgery, The Hospital for Sick Children, Toronto, ON, Canada M5G 1X8; Developmental and Stem Cell Biology Program, Peter Gilgan Centre for Research and Learning, The Hospital for Sick Children, Toronto, ON, Canada M5G 0A4; Division of General and Thoracic Surgery, The Hospital for Sick Children, Toronto, ON, Canada M5G 1X8; Developmental and Stem Cell Biology Program, Peter Gilgan Centre for Research and Learning, The Hospital for Sick Children, Toronto, ON, Canada M5G 0A4; Division of General and Thoracic Surgery, The Hospital for Sick Children, Toronto, ON, Canada M5G 1X8; Department of Surgery, University of Toronto, Toronto, ON, Canada M5T 1P5

**Keywords:** exosomes, fetal therapy, regenerative therapy, congenital diaphragmatic hernia, stem cells, inflammation

## Abstract

Disruption of developmental processes affecting the fetal lung leads to pulmonary hypoplasia. Pulmonary hypoplasia results from several conditions including congenital diaphragmatic hernia (CDH) and oligohydramnios. Both entities have high morbidity and mortality, and no effective therapy that fully restores normal lung development. Hypoplastic lungs have impaired growth (arrested branching morphogenesis), maturation (decreased epithelial/mesenchymal differentiation), and vascularization (endothelial dysfunction and vascular remodeling leading to postnatal pulmonary hypertension). Herein, we discuss the pathogenesis of pulmonary hypoplasia and the role of microRNAs (miRNAs) during normal and pathological lung development. Since multiple cells and pathways are altered, the ideal strategy for hypoplastic lungs is to deliver a therapy that addresses all aspects of abnormal lung development. In this review, we report on a novel regenerative approach based on the administration of extracellular vesicles derived from amniotic fluid stem cells (AFSC-EVs). Specifically, we describe the effects of AFSC-EVs in rodent and human models of pulmonary hypoplasia, their mechanism of action via release of their cargo, including miRNAs, and their anti-inflammatory properties. We also compare cargo contents and regenerative effects of EVs from AFSCs and mesenchymal stromal cells (MSCs). Overall, there is compelling evidence that antenatal administration of AFSC-EVs rescues multiple features of fetal lung development in experimental models of pulmonary hypoplasia. Lastly, we discuss the steps that need to be taken to translate this promising EV-based therapy from the bench to the bedside. These include strategies to overcome barriers commonly associated with EV therapeutics and specific challenges related to stem cell-based therapies in fetal medicine.

## Graphical Abstract

**Graphical Abstract:**
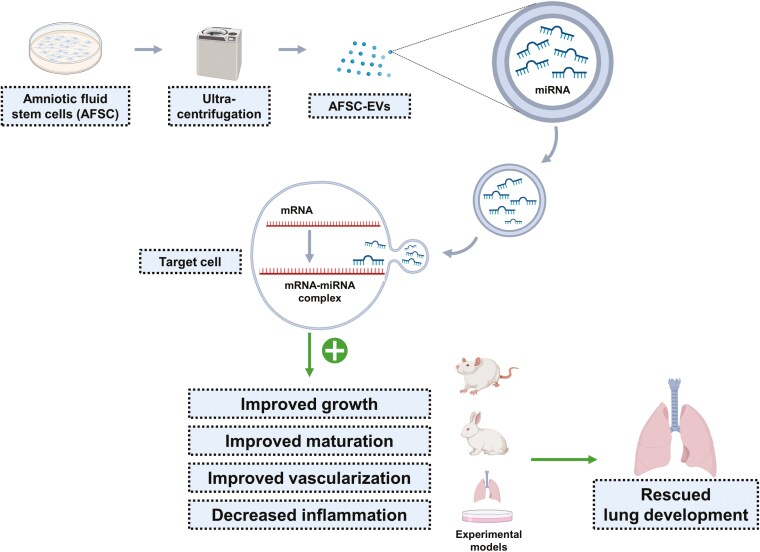

**Significance statement**

Lung underdevelopment is associated with high mortality and severe complications in survivors. The hallmark of this disease regardless of the underlying cause is characterized by impaired lung growth, maturation, and vessel formation. The optimal therapy is one that addresses all aspects of lung development before birth. Extracellular vesicles (EVs) are droplets released by all cells for communication purposes. Herein, we describe how antenatal administration of stem cell-derived EVs rescues lung development in experimental models via the release of factors that are missing in underdeveloped lungs. Moreover, we discuss the steps necessary to take this promising therapy to the bedside.

## Pathogenesis of pulmonary hypoplasia

**Figure 1.**
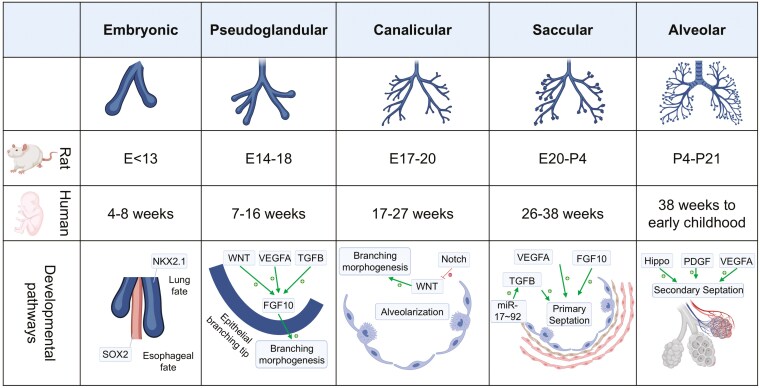
Stages of lung development in rats and humans.

Lung development is a finely orchestrated process whereby fetal pulmonary buds grow and mature throughout gestation until early childhood.^1^ This process requires specialized cell populations, such as airway epithelial, mesenchymal, endothelial, and immune cells, and is articulated through 5 stages (embryonic, pseudoglandular, canalicular, saccular, and alveolar).^1-3^ (**Figure 1**) Patterning and coordination of these cell populations with mature epithelial airway cells in close proximity to capillaries surrounded by mesenchyme, ultimately leads to the establishment of the main function of the organ, that is gas exchange.^4,5^ During the embryonic stage, the primitive lung bud, expressing NKX2.1, separates from the primitive esophageal bud, expressing SOX2, a process that occurs from 4 to 8 weeks in human fetuses.^4,6,7^ Subsequently, during the pseudoglandular stage lasting until the 16th week of gestation, the epithelial lung bud invades the mesenchyme, where extensive, dichotomous proliferation occurs in a process referred to as branching morphogenesis.^4^ This process is dependent on various signaling pathways, such as fibroblast growth factors (FGF), vascular endothelial growth factor (VEGF), WNT, and transforming growth factor beta (TGFB) signaling. FGF10 is a guiding cue for the proliferating epithelium, which is further promoted by VEGF expression leading to the development of supportive vascular tissue that supplies branching epithelial tips.^8-10^ WNT-signaling activates NKX2.1 expression and enables the primordial lung bud to sprout. TGFB-signaling controls cell differentiation, proliferation, and motility, and its abnormal expression results in lung branching morphogenesis defects.^11-14^ During the canalicular stage that spans from 17 to 27 weeks of gestation in humans, distal epithelial airway cells mature with two distinct cell types: alveolar type I (ATI) and alveolar type II cells: ATI cells make most of distal airway epithelial cells and are responsible for postnatal gas-exchange; ATII cells process lipoproteins to later secrete surfactant and are precursors of ATI cells, postnatally.^4,15,16^ During this developmental stage, physical forces related to fetal breathing movements and transpulmonary pressure are also critical to initiate and maintain lung branching morphogenesis and epithelial cell plasticity.^17,18^ During the saccular and alveolar stages, ATII cells accelerate producing surfactant, the mesenchyme thins out, and the capillary bed expands extensively to prepare the fetus for gas exchange and postnatal adaptation.^19,20^

When lung developmental processes are altered, the fetal lung tissue fails to properly mature and grow. This could lead to alterations to localized areas of the lung giving rise to lesions such as pulmonary sequestration and congenital pulmonary airway malformation, or it could involve the whole lung that becomes hypoplastic (pulmonary hypoplasia).^4,5^ Although these events are rare, pulmonary hypoplasia is detected in up to 20% of perinatal autopsy studies.^21^ In fact, pulmonary hypoplasia can be present as a result of several conditions, including congenital diaphragmatic hernia (CDH) and oligohydramnios. CDH is a rare birth defect characterized by incomplete closure of the diaphragm and herniation of fetal abdominal organs into the thoracic cavity leading to arrested development of the lungs that become hypoplastic.^22,23^ Compared to normal, CDH hypoplastic lungs have fewer branches and alveoli (decreased growth), undifferentiated epithelium and mesenchyme (impaired maturation), and fewer and muscularized lung vessels (vascular remodeling) leading to postnatal pulmonary hypertension.^22^ There is emerging evidence that arrested lung development may be driven by inflammatory processes in the fetal lung with macrophage enrichment.^24-26^

On the other hand, oligohydramnios is defined as decreased amniotic fluid volume, often caused by leakage through ruptured membranes that results in fetal pulmonary hypoplasia. The phenotypical changes observed in oligohydramnios lungs are similar to those also seen in CDH. Both conditions have decreased lung dimensions and impaired lung branching morphogenesis, altered epithelial and mesenchymal cell differentiation with fewer ATI or myofibroblast progenitor cells, and impaired vascularization.^15-18,27-33^ For both CDH and oligohydramnios fetuses, the pathogenesis of pulmonary hypoplasia remains elusive. Nonetheless, it is known that the amniotic fluid itself contains growth factors, proteins, lipids, RNA species among other bioactive molecules that also contribute to normal lung development.^34^

## Role of microRNAs in regulating lung development

Among all factors implicated in normal lung development, special consideration has been given to microRNAs (miRNAs) in the last decade. miRNAs are small non-coding RNAs that consist of ~22 nucleotides.^35^ They downregulate gene expression by binding to messenger RNAs and causing their degradation or by inhibiting translation.^36^ miRNAs are recognized to play a critical role in organogenesis, including fetal lung development, where they regulate key signaling molecules like FGF, TGFB, and WNT. There are several miRNAs and miRNA clusters (a group of miRNA genes that are processed from a single primary transcript) that regulate processes of lung growth, maturation, and vascularization. The miR17~92 cluster consists of polycistronic miRNA genes encoding 15 miRNAs that control FGF-10-mediated branching morphogenesis, lung epithelial cell proliferation, differentiation, and apoptosis.^37-41^ Targeted deletion of the miR17~92 cluster in transgenic mice causes fatal bilateral pulmonary hypoplasia and ventral septal defect.^41^ This miRNA cluster has also been reported to be downregulated in human preterm babies that develop bronchopulmonary dysplasia, a severe form of chronic lung disease that typically affects neonates that are born prematurely.^42^ Moreover, miR-200 and miR-93-5p control branching morphogenesis and epithelial plasticity during human fetal lung development via modulation of TGFB.^43-45^ miR-200b deficient transgenic mice have features of pulmonary hypoplasia, including dysregulated epithelial cell differentiation and thicker alveolar walls.^46^ In addition, miR-142 is involved in WNT signaling during lung development as it has been reported to regulate proliferation of mesenchymal progenitor cells.^47^

There has been growing evidence that experimental CDH and human fetuses with pulmonary hypoplasia secondary to CDH have missing or dysregulated miRNA expression. In studies investigating fetuses with CDH treated with a fetoscopic procedure called fetoscopic endotracheal occlusion (FETO), researchers reported differential expression of miR-379-5p, miR-889-3p, miR-223-3p, miR-503-5p, miR-200b, and miR-10 between survivors and non-survivors.^48,49^ Moreover, tracheal fluid of patients undergoing FETO showed upregulation of some of the miR17~92 cluster (miR-16, miR-17, miR-18, miR-19b, and miR-20a) compared to age-matched controls.^50^ A study investigating the expression of circulating miRNAs in blood samples drawn within 24 hours after birth reported differential expression levels of miRNAs let-7b/c, miR-1307-3p, miR-185-3p, miR-8084, miR-331-3p, and miR-210-3p between CDH babies that either died or developed chronic lung disease up to 28 days after birth compared to survivors that did not develop lung disease.^51^ Examination of fetal CDH lung autopsy specimens indicated dysregulated miR-449a patterning and over-expression of its downstream target N-MYC that is known to be associated with human CDH.^52,53^ Lastly, miRNA dysregulation has also been reported in experimental CDH. This includes the dysregulation of miR-130a-5p with impaired *Foxa2* expression leading to impaired lung branching in the rat model of CDH.^54^ Another study reported a marked decrease in miR-33, a miRNA that regulates epithelial–mesenchymal interactions, Wnt signaling pathways, and macrophage immuno-metabolic response.^55,56^

## Use of amniotic fluid stem cells extracellular vesicles as a regenerative therapy

Since miRNAs are pivotal during lung organogenesis, addressing the missing or dysregulated miRNA levels in fetal hypoplastic lungs could be a strategy to foster normal lung development. Extracellular vesicles (EVs), lipid-bound particles secreted by cells, transport a diverse array of miRNAs and play crucial roles in intercellular communication and signaling, both in normal physiological conditions and during pathological states.^57,58^ EVs transport genetic material like miRNAs and bioactive proteins to target cells, activating biological responses.^59^ Due to this ability, EVs are being explored as therapeutic agents for conditions characterized by deficiencies or dysregulation in multiple molecules and pathways.^58^ These include a variety of conditions such as bronchopulmonary dysplasia, retinopathy of prematurity, necrotizing enterocolitis, or perinatal brain injury, as well as myocardial infarction.^60-65^

There is consensus that the ideal therapy for pulmonary hypoplasia would be delivered antenatally and would address all aspects of impaired lung development, that is impaired lung growth and branching morphogenesis, altered epithelial and mesenchymal cell maturation, and impaired vascularization with vascular remodeling. To this end, EVs derived from amniotic fluid stem cells (AFSCs) and mesenchymal stromal cells (MSCs) have shown promise. AFSCs have been reported to exhibit significant capacity to repair lung injury and induce lung growth in experimental models of CDH.^66-68^ This is due to their ability to differentiate into lung cell lineages and promote alveolar wound healing, epithelial cell differentiation, and lung homeostasis via paracrine signaling.^69^ In a fetal rat model of CDH, Pederiva et al demonstrated that AFSC administration rescued lung growth back to normal levels.^67^ Using the same model of CDH, Di Bernardo et al proved similar benefits with branching morphogenesis and lung epithelial maturation following administration of AFSC conditioned medium.^66^ Tzanetakis et al demonstrated that administering AFSCs to primary epithelial cells extracted from rat fetal hypoplastic lungs restored their tissue balance. This was achieved by reducing the cellular endoplasmic reticulum stress response and apoptosis while enhancing both cell proliferation and migration capabilities.^68^ Moreover, DeKoninck et al reported that intratracheal AFSC administration using the fetal rabbit CDH model accelerated the beneficial effects of tracheal occlusion in rescuing branching morphogenesis and alveolarization.^70^

As the EV cargo reflects the molecular profile of their parental cells, AFSC and MSC-EVs have been tested in several in-vitro, ex-vivo, and in-vivo rodent and human disease models of pulmonary hypoplasia (Table 1)^16,24,33,71-79^ In these models, the authors reported that antenatal administration of AFSC-EVs restores growth, maturation, and vascularization in fetal hypoplastic lungs secondary to CDH.^16,33,73-75,77^ These beneficial effects were obtained not only in the pseudoglandular stage of lung development but also during the canalicular and saccular stages that are translationally relevant timepoints for potential antenatal CDH therapy.^16^ The effects of AFSC-EVs on lung growth were evidenced by the increased number of airspaces and restoration of key factors involved in branching morphogenesis, such as *Fgf10*, *Vegfa*, *Flt1*, and *Kdr*.^73^ Restoration of lung maturation in hypoplastic CDH lungs was indicated by increased lung differentiation of epithelial (ATI, ATII, club, ciliated epithelial, and pulmonary neuroendocrine cells) and mesenchymal cells (lipofibroblasts).^16,73^ Some of these effects were also observed during the alveolar stage of lung development, as shown in the surgical model of CDH in fetal rabbits, where alveolar formation in this species occurs prenatally.^73^ Lung vascular regeneration following AFSC-EV administration was characterized by restoration of vascular density, expression of key angiogenic markers, such as *Vegfa*, *Vegfr1,* and *Vegfr2*, and attenuation of pulmonary vascular remodeling.^33^ Beyond structural and molecular improvements, Figueira et al also demonstrated that intra-amniotic administration of AFSC-EVs in fetal rats with CDH improved lung mechanics, as evidenced by the improvement in lung resistance, elastance, and compliance, as well as reduced lung collagen deposition.^76^

**Table 1.** Overview of experimental studies using AFSC-EVs or MSC-EVs as a treatment for pulmonary hypoplasia.

Study	Source	Model	Outcome measures	Summary
Zhaorigetu et al ^71^	Bone marrow-derived human MSC-EVs	- In vitro injured (nitrofen) human pulmonary artery endothelial cells (HPAEC) co-cultured with MSC-EVs - In vivo nitrofen-exposed fetal rats with CDH treated with MSC-EVs	- Cell viability - Protein analysis of endothelial cell dysfunction markers - Rat pulmonary artery contractility and relaxation	MSC-EV administration improved: - Injured HPAEC cell viability and protein expression of LOX-1, NOX-2, NF-kB, p-PPARy, ET-1, p-ENOS - CDH pulmonary artery contractility
Monroe et al ^72^	Bone marrow derived human MSC-EVs	- In vivo nitrofen-exposed fetal rats with CDH treated with MSC-EVs	- Elastin, collagen I, LOX, and MMP-9 expression in the main pulmonary artery - Medial wall thickness and collagen fibril width via transmission electron microscopy - Mechanical characterization of vessels via myography	MSC-EVs administration led to: - Decreased elastin composition, - Inhibition of extracellular matrix remodeling enzymes (LOX, and MMP-9) and rescue of medial wall thickness.
Antounians et al^73^	Rat AFSC-EVs GMP-grade human AFSC-EVs	- Nitrofen-exposed fetal rat lung explants, primary lung epithelial cells, and organoids - Surgical fetal rabbit model of CDH with tracheal occlusion and intratracheal AFSC-EV administration - Nitrofen injured human A549 and human fetal alveolar epithelial cells	- Lung growth and branching (terminal buds, lung surface, mean linear intercept, gene expression *Fgf10*) - Fetal lung vascularization (gene expression *Vegfa*, *Flt1*, *Kdr*) - Fetal lung maturation (SPC, SOX9) - Mesenchymal maturation (Bmp2, Bmp4)	Rat AFSC-EVs improved: - Terminal bud count, lung surface area, and expression of Fgf10, Vegfa, Flt1, Kdr, and Sox9, SPC in fetal hypoplastic rat lung explants - Proliferation and viability of rat primary lung epithelial cells - Ki67, SPC, and CC10 expression in rat fetal lung organoids - Number of alveoli, alveolar wall thickness, and expression of PLIN2, BMP2, BMP4, and ID1 in rabbit CDH hypoplastic lungs - Proliferation and viability rates in human A549 and human fetal alveolar epithelial cells
Khalaj et al 2022^16^	Rat AFSC-EVs	- Nitrofen-exposed rat fetal lung explants	- Number of airspaces - Gene and protein expression for various markers of fetal lung branching morphogenesis and epithelial and mesenchymal maturation	Rat AFSC-EV improved: - Number of airspaces - Expression of lung branching morphogenesis markers (eg, *Fgf10*, *Nrp1*, *Ctnnb1*) - Expression of lung epithelial and mesenchymal maturation markers (eg, PDPN, SPC, CC10, FOXJ1, etc.)
Khalaj et al^74^	Rat AFSC-EVs GMP-grade human AFSC-EVs	- Ex vivo nitrofen-exposed rat fetal lung explants - In vivo nitrofen CDH rat model - Ex vivo fetal human pulmonary hypoplasia model induced by NSC23766 administration	- Number of airspaces - Gene and protein expression for autophagy markers - Validation of miR-17 ∼ 92 inhibition via gene expression of *Bmpr2*, *Hdac4*, and *Atg7*	Rat AFSC-EVs improved: - airspace density - expression of autophagy markers (BECN1, ATG5, and SQSTM1) GMP-grade human AFSC-EVs rescued autophagy levels (BECN1, ATG5, SQSTM1)
Blundell et al^75^	Rat AFSC-EVs	- In vivo nitrofen CDH rat model	- Number of airspaces - Fetal lung inflammation (Gene expression *Tnfa*) - Fetal brain inflammation (protein and gene expression Iba1 + microglia, TNFa, *Il1b*)	Rat AFSC-EVs improved: - Lung branching morphogenesis - Fetal lung inflammation (*Tnfa*) - Fetal neuroinflammation (Iba-1 + microglia, TNFa, Il1b)
Figueira et al^33^	Rat and human AFSC-EVs	- Compression model of fetal rat and human lung explants - In vivo nitrofen CDH rat model	- Fetal lung vascularization (gene and protein expression of Vegfa, Vegfr1/2, Cd31, Enos, Epas1, Gata2, Ang1, Tie2) - Hippo signaling pathway (Gene expression of *Yap*, *Taz*, etc.)	Rat and human AFSC-EVs improved key angiogenic factors. Rat AFSC-EVs improved expression of angiogenic factors and Hippo signaling
Antounians et al^24^	Rat AFSC-EVs	- In vivo nitrofen CDH rat model	- Number of airspaces - Single nucleus RNA sequencing of fetal rat lungs - Fetal lung macrophage enrichment (immunofluoresence CD68, TNFa; flowcytometry for CD68, ADGRE-1, and CD43)	Rat AFSC-EVs: - Dampened multilineage inflammatory signaling in vivo in fetal rat lungs. - Rescued fetal rat lung macrophage enrichment in vivo back to control - In vivo inhibition of macrophages via GW2580 improved airspace density
Figueira et al^76^	Rat AFSC-EVs	- In vivo nitrofen CDH rat model, ventilation of pups at birth	- Number of airspaces - Collagen deposition (picrosirius red) - Ventilation parameters	Rat AFSC-EVs improved: - Airspace density - Collagen deposition - Lung mechanics (compliance, resistance, tissue damping, elastance, tissue elastance)
Doktor et al^45^	Rat AFSC-EVs	- Ex vivo rat model of oligohydramnios	- Number of airspaces - Lung branching morphogenesis and progenitor cell expression (Gene expression of *Fgf10*, *Nrp1*, *Ctnnb1, Sox2, Sox9*) - Transforming growth factor beta signaling pathway (TGFB)	Rat AFSC-EVs - Improved airspace density (RAC, MLI) - Improved lung branching morphogenesis (*Fgf10*, *Nrp1*, *Ctnnb1*) and lung progenitor cell expression. - Mediated at least in part via miR-93-5p release and the modulation of TGFB signaling pathway
Doktor et al^77^	Rat AFSC-EVs	- Ex vivo nitrofen and bisdiamine exposed fetal mouse lung explants	- Number of airspaces - Gene expression of fetal lung vascularization (Cd31, Enos) - Gene and protein expression of fetal lung inflammation (TNFa, *Il1b*)	Rat AFSC-EVs improved: - lung branching morphogenesis (MLI), - key angiogenic factors (Cd31, Enos) - fetal lung inflammation (TNFa, Il1b)

MSCs and their EVs (MSC-EVs) have also been reported to exhibit regenerative potentials when administered in utero to fetal hypoplastic lungs secondary to CDH (Table 1).^66,71,72,80-84^ In particular, MSC-EV administration has been reported to exert beneficial effects on the endothelium of fetal hypoplastic lungs.^71,72^ In this study, the authors showed that administration of MSC-EVs led to decreased reactive oxygen species production in vitro using stressed human pulmonary artery endothelial cells. Similarly, in vivo administration of MSC-EVs in an experimental rodent model of CDH improved contractility of the pulmonary artery and decreased the medial wall thickness suggesting improved vascular remodeling.^71,72^

**Figure 2.**
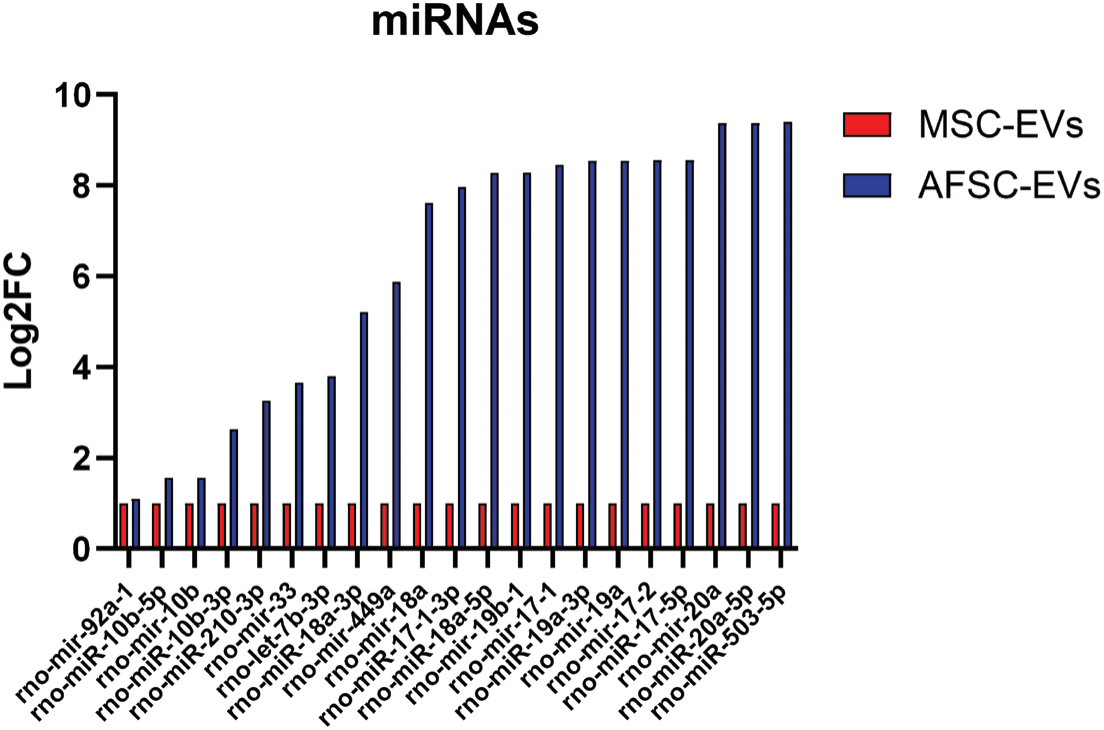
Selected miRNAs differentially expressed within AFSC-EVs compared to MSC-EVs (adapted from Antounians et al. Sci Transl Med 2021).

The AFSC-EV mechanism of action is classically ascribed to the release of the EV cargo content.^58^ Experimental studies on CDH fetal hypoplastic lungs reported that AFSC-EV effects were found to be at least in part due to the release of small RNA species contained in the EV cargo.^73,74^ The authors detected fluorescently labeled RNA cargo in the lung parenchyma and observed that administration of AFSC-EVs whose RNA cargo was enzymatically digested did not rescue lung development.^73^ The role of the RNA cargo was further confirmed by miRNA inhibition studies, where AFSCs transfected with antagomirs against miR-17 and -20a produced EVs that were not able to improve branching morphogenesis in rat fetal hypoplastic lungs.^74^ Small RNA sequencing analysis showed that the AFSC-EV cargo was highly enriched with miRNAs responsible for mediating the expression of genes involved in lung development, such as the miR17 ∼ 92 family cluster, miR-200, miR-379-5p, miR-223-3p, miR-503-5p, miR-93-5p, miR-10, let-7b/c, miR-185-3p, miR-331-3p, miR-210-3p, miR-449a, miR-130a-5p, or miR-33.^45,48,49,51,52,54,55,59,73,85^ Interestingly, Antounians et al found that some of these miRNAs were differentially expressed when compared to the RNA cargo of MSC-EVs (Figure 2).^73^ Other studies demonstrated that MSC-EV regenerative effects are secondary to some of the same miRNAs, such as miR-223/142 (inhibition of the NLRP3 inflammasome, immunomodulatory effects on dendritic cells) or let-7c (promotes cerebral angiogenesis).^86-88^

As the EVs cargo also contains other bioactive molecules, further research has evaluated their potential role in mediating regenerative effects of AFSC- and MSC-EVs. Proteomic analysis of the AFSC-EV cargo revealed the presence of proteins involved in miRNA stabilization and EV structure and function.^24,73^ Several studies have found functional proteins within MSC-EVs that have immunomodulatory and regenerative effects, including Programmed death-ligand 1 (PD-L1), Galectin-1, TGF-beta, VEGF, Neprilysin, or Platelet-derived growth factor D (PDGF-D).^89^ Further research will determine if other EV cargo compounds, such as lipids or other non-coding RNA species contribute to the observed regenerative effects.

Towards clinical translation, 2 studies tested the effects of EVs derived from human AFSCs isolated with good manufacturing practices (GMP) on human alveolar epithelial cells (A549), human fetal pulmonary alveolar epithelial cells (HPAEpiC), and human fetal lung explants.^73,74^ In these models, normal lung cells and tissue were stressed with either nitrofen or NSC23766, which are inhibitors of the retinoic acid pathway. Administration of human AFSC-EVs restored primary lung cell proliferation and viability, as well as lung branching morphogenesis.^73,74^ Moreover, using both human and rat models of pulmonary hypoplasia, Khalaj et al. reported that the administration of AFSC-EVs also rescued autophagy, an important biological process involved in cell homeostasis and lung development.^74^ Further, Zhaorigetu et al tested human-derived MSC-EVs on nitrofen-injured human pulmonary artery endothelial cells and reported improved endothelial cell dysfunction and viability.^71^

## Anti-inflammatory effects of AFSC-EVs

**Figure 3.**
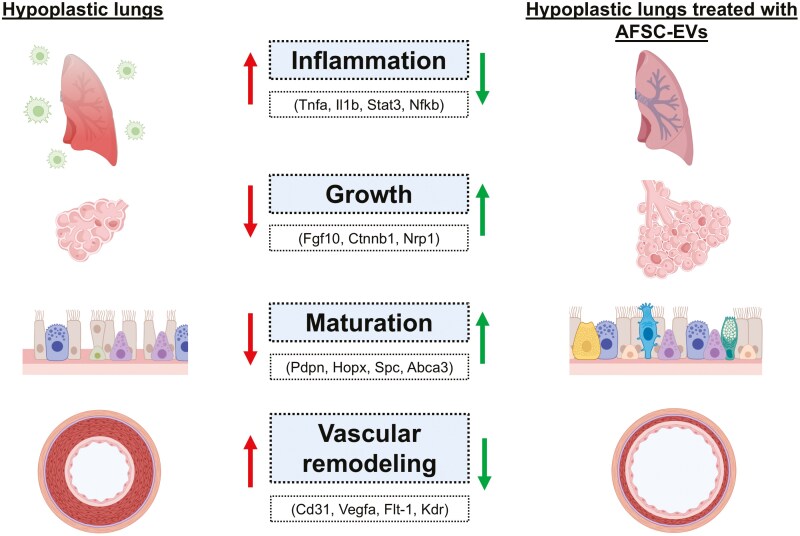
Effects of antenatal AFSC-EV administration on hypoplastic lungs secondary to CDH.

To better understand AFSC-EV effects on the hypoplastic lung at a cellular and molecular level, Antounians et al conducted transcriptomics analysis and compared control fetal lungs, hypoplastic lungs secondary to CDH treated with saline, and hypoplastic lungs secondary to CDH treated with AFSC-EVs.^24^ When experimental CDH lungs were treated with AFSC-EVs, single nucleus RNA-sequencing, flowcytometry, and immunofluorescence confirmed dampened multilineage inflammatory signaling and rescued macrophage density.^24^ As macrophages may be the drivers of this multilineage inflammatory signature and arrested fetal lung development, the authors antagonized macrophage survival, proliferation, and function via inhibition of the colony stimulating factor 1 receptor (Csf1r) that is expressed by all macrophages. Remarkably, Csf1r inhibition resulted in less severe pulmonary hypoplasia.^24^ These data suggested that AFSC-EVs hold anti-inflammatory and regenerative properties on CDH fetal hypoplastic lungs (Figure 3). This is in line with studies in other experimental disease models, such as necrotizing enterocolitis, where administration of AFSCs, AFSC conditioned medium, or AFSC-EVs downregulated inflammatory responses, decreased apoptosis, reduced intestinal epithelial cell damage, improved survival, and increased epithelial proliferation and stem cell activity.^62,90-94^ Further studies using novel spatial biology techniques will enable the creation of a more comprehensive cellular map of events that contribute to organ injury and illustrate how AFSC-EVs exert anti-inflammatory effects and promote regeneration.^95^

Similarly, MSCs and their EVs have shown anti-inflammatory effects in bronchopulmonary dysplasia (BPD), a neonatal disease characterized by extensive lung inflammation and macrophage involvement.^96^ In these studies, administration of MSCs and their EVs improved lung alveolarization and downregulated inflammatory processes by immunomodulating pro-inflammatory “M1-like” into reparative “M2-like” macrophages.^97,98^

Further proof of AFSC-EV anti-inflammatory properties is their effects on the brain of fetuses with CDH, which is known to have a neuroinflammatory signature with loss of progenitor cells, neurons, and oligodendrocytes.^99^ In an in vivo rat model of CDH, intra-amniotically injected AFSC-EVs reached the fetal brain, attenuated *Tnfa* and *Il1b* expression, and reduced activated microglia density in the sub-granular zone, which is the neurogenic niche of the brain.^75^ This is in line with a recent study that reported how AFSC-EVs are able to decrease the expression of pro-inflammatory markers in dendritic cells, leading to the suppression of autoimmune responses in a mouse model of multiple sclerosis.^100^

## Challenges inherent to the translation of AFSC-EVs to clinical application

**Figure 4.**
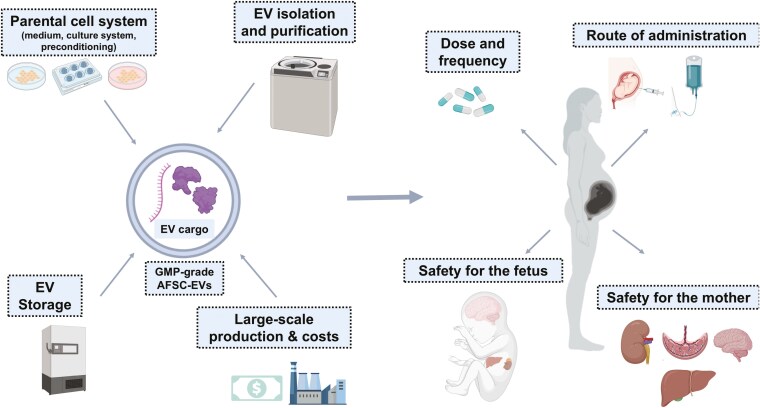
Challenges inherent to the translation of AFSC-EVs to clinical application.

Given the promising preclinical results obtained with fetal AFSC-EV administration in various experimental models, evaluating this promising novel therapeutic approach in clinical practice is necessary. Although the field of EV therapeutics is noticeably young, there are already several applications that are being tested or have been tested in human patients with numerous medical conditions. This includes 18 clinical trials registered on ClinicalTrials.gov that are recruiting patients to be treated with EV therapies for conditions, such as acute respiratory distress syndrome (ARDS), burn wounds, or inflammatory bowel diseases (**Table 2**). Moreover, the first safety and efficacy trials reporting EVs as therapeutic strategies in human patients with malignant neoplastic, respiratory, and neurodegenerative diseases have been published.^123^ Although these studies had significant heterogeneity, serious adverse effects were rarely reported.^124^ Despite the promising advances in the translation of EVs to the clinical realm, there are still challenges that need to be addressed and overcome before AFSC-EVs can be administered to fetuses with pulmonary hypoplasia. This is in part due to common challenges that EV therapeutics are associated with and in part to the type of diseases being addressed (**Figure 4**).

**Table 2.** Ongoing clinical trials assessing extracellular vesicles as a therapeutic option registered on ClinicalTrials.gov.

Title	Description	Clinical Trials ID
Phase IIa multi-center prospective, randomized trial to evaluate the safety and efficacy of topical PEP-TISSEEL for diabetic foot ulcers (DFU)^101^	- Platelet-derived EVs loaded with fibrin sealant^102^ - Derived via serial filtration, enucleation and centrifugation^102^ - Prospective randomized trial to evaluate safety and efficacy for diabetic foot ulcer	**NCT06319287**
ExoFlo™ infusion for post-acute COVID-19 and chronic post-COVID-19 syndrome^103^	- EVs derived from allogeneic bone marrow mesenchymal stem cell (MSC)^104,105^ - Phase I/II clinical trial to assess the safety and efficacy of MSC-EVs in post-acute COVID-19 and chronic post-COVID-19 syndrome	**NCT05116761**
Intra-ovarian injection of MSC-EVs in idiopathic premature ovarian failure^106^	- Safety and feasibility study of intra-ovarian injection of bone marrow MSC-EVs in idiopathic premature ovarian failure patients	**NCT06202547**
Study of ExoFlo for the treatment of medically refractory ulcerative colitis^107^	- EVs derived from allogeneic bone marrow mesenchymal stem cell (MSC)^104^ - Safety and feasibility phase I trial of intravenous (iv) MSC-EVs in patients with medically refractory ulcerative colitis	**NCT05176366**
Study of ExoFlo for the treatment of medically refractory Crohn’s disease^108^	- EVs derived from allogeneic bone marrow mesenchymal stem cell (MSC)^104^ - Safety and feasibility phase I trial of iv MSC-EVs in patients with medically refractory Crohn’s disease	**NCT05130983**
Study of ExoFlo for the treatment of perianal fistulas^109^	- EVs derived from allogeneic bone marrow mesenchymal stem cell (MSC)^104^ - Safety and efficacy phase IB/IIA, multicenter, placebo-controlled, randomized controlled trial for the treatment of perianal fistulizing Crohn’s disease	**NCT05836883**
Safety and efficacy of MSC-EVs in the prevention of BPD in extremely preterm infants (EVENEW)^110^	- Large EVs derived from umbilical cord MSCs^111^ - Phase I/II safety and efficacy trial evaluating the application of intratracheal umbilical cord-derived MSC-EVs for the treatment of bronchopulmonary dysplasia (BPD) in extremely preterm neonates (23 up to 28 weeks, 500-1500*g*)	**NCT06279741**
MSC EVs in dystrophic epidermolysis bullosa^112^	- Allogenic MSC-EVs - Phase 1/2A, non-randomized trial to assess safety and efficacy of MSC-EVs to treat lesions in patients with Epidermolysis Bullosa	**NCT04173650**
Extracellular vesicle treatment for acute respiratory distress syndrome (ARDS) (EXTINGUISH ARDS)^113^	- EVs derived from allogeneic bone marrow mesenchymal stem cell (MSC)^104^ - Phase III, multicenter, randomized, double-blinded, placebo-controlled study assessing the treatment of Acute Respiratory Distress Syndrome (ARDS) with MSC-EVs	**NCT05354141**
Expanded access for use of bmMSC-derived extracellular vesicles in patients with COVID-19-associated ARDS^114^	- EVs derived from allogeneic bone marrow mesenchymal stem cell (MSC)^104^ - Expanded access protocol for all patients that are not eligible for phase III trial: **NCT05354141**	**NCT04657458**
Autologous serum-derived EV for venous trophic lesions not responsive to conventional treatments (SER-VES-HEAL)^115^	- Serum-derived EVs - Phase II trial assessing the efficacy of serum-derived EVs for the treatment of venous trophic lesions not responsive to conventional treatments	**NCT04652531**
Safety evaluation of intracoronary infusion of extracellular vesicles in patients following coronary stent implantation^116^	- Safety study of intra-coronary administration of EVs derived from blood applied after percutaneous coronary intervention for coronary stent implantation	**NCT04327635**
Safety and efficacy of stem cell small extracellular vesicles in patients with retinitis pigmentosa^117^	- Single, intravitreal injection of GMP-grade bone marrow-derived small MSC-EVs to assess safety and efficacy for retinitis pigmentosa	**NCT06242379**
Randomized, controlled, multicenter study of extracellular vesicles from human adipose tissue promoting wound healing^118^	- Adipose tissue-derived extracellular vesicles - Phase II trial assessing the efficacy of EVs derived from adipose tissue in patients with full-layer skin ulcers	**NCT06253975**
Bone marrow mesenchymal stem cell-derived extracellular vesicles infusion treatment for ARDS (EXIT-ARDS)^119^	- EVs derived from allogeneic bone marrow mesenchymal stem cell (MSC)^104^ - Safety and efficacy phase I/II trial to assess iv administration of bone marrow-derived MSC-EVs for ARDS	**NCT05127122**
Treatment of non-ischemic cardiomyopathies by intravenous extracellular vesicles of cardiovascular progenitor cells (SECRET-HF)^120^	- Phase I/II trial assessing safety and efficacy of EV enriched secretome obtained from cardiovascular progenitor cells for non-ischemic dilated cardiomyopathy	**NCT05774509**
Extracellular vesicles from mesenchymal cells in the treatment of acute respiratory failure^121^	- Phase I/II safety and efficacy trial (randomized, double-blind, placebo-controlled) evaluating MSC-EVs in patients with ARDS secondary to COVID-19 or other etiologies	**NCT06002841**
Safety of extracellular vesicles for burn wounds^122^	- Phase I/II trial to assess safety and efficacy of topical, allogenic MSC-EV administration to deep second-degree burn wounds	**NCT05078385**

Ideally, to ensure a standardized clinical application, administered EVs should carry a homogeneous cargo content to eliminate or at least reduce batch-to-batch variability. The EV cargo might be influenced by various factors, including the composition of the parental cell medium, the parental cell culture system (2D vs 3D systems), parental cell preconditioning with factors such as cytokines or hypoxia, EV isolation and purification techniques, as well as EV storage conditions.^125,126^ Some of these challenges may provide opportunities to improve EV quality and to tailor them for different therapeutic applications. A way to minimize EV cargo variability is by immortalizing parental cells. This approach has been reported in several studies, which have confirmed that immortalized cell lines reduce cargo heterogeneity and batch-to-batch variability, with improvement of manufacturing costs and optimization of therapeutic efficiency.^127-130^ The most practical solution would involve biobanking AFSCs from the amniotic fluid of healthy pregnancies, separating AFSC-EVs in a GMP fashion, and utilizing them as a heterologous therapy. Since no standardization exists for the isolation and purification of EVs, several techniques based on differential sedimentation, solubility, and size-exclusion separation have been reported for AFSC-EVs.^79^ In a study comparing AFSC-EV isolation techniques, ultracentrifugation was one of the modalities that separated the small-size EVs that were effective in preventing lung epithelial cell death.^79^ Interestingly, most clinical studies investigating the therapeutic effects of EVs in human trials use ultracentrifugation to isolate EVs.^123^ Another challenge for EV therapeutics relates to the type of storage strategies.^131^ A systematic review of the literature revealed that temperature and repeated freeze-thaw cycles may influence EV concentration and purity leading to fused and artifactual EVs.^131^ Achieving a significant clinical effect often necessitates large doses of EVs, far surpassing the yield of natural vesicles produced by cells. Thus, there is a critical need for reliable and scalable approaches to generate clinical-grade EVs. However, those methods must be cost-effective as pharmaceutical EV derivation in a financially feasible manner is challenging.^132^

Besides EV-specific issues to address, there are also challenges related to the type of disease that needs to be treated. This is particularly true in regenerative fetal medicine, as none of the ongoing trials recruiting patients include study subjects under the age of 18 (**Table 2**). The main challenges are related to EV dose and frequency, route of administration, and safety for the fetus and the mother (**Figure 4**). In particular, establishing the optimal EV dosing and route of administration may be challenging due to the limited understanding of EV pharmacokinetics and pharmacodynamics.^133^ In rodent models of CDH, the dose of AFSC-EVs to be administered was established based on dose-response experiments that determined a therapeutic dosage of 7.6 × 10^9^ EVs in 100 µL of saline per rat fetus.^24,73,74^ However, studies in large animal models are needed to establish the therapeutic dose for human fetuses with CDH. In experimental rat and rabbit in vivo models of CDH, one single dose administration was enough to obtain the desired effects on fetal lungs.^24,73,74^ As EVs have a relatively short half-life and bioactivity, studies in large animals will also have to address whether a single dose is enough to rescue lung development.^134,135^ Even though some studies in humans used single doses of therapeutic EVs, the majority of trials applied multiple dosages.^123^ The optimal route of administration to deliver AFSC-EVs in fetuses with pulmonary hypoplasia secondary to CDH remains unknown. Experimental studies in rats and rabbits have shown promise with both intra-amniotic and intra-tracheal injections of AFSC-EVs. On the one hand, pregnant mothers carrying fetuses with CDH typically undergo amniocentesis, hence AFSC-EVs could be administered via this route at the same time.^136^ On the other hand, the intra-tracheal route is likely the most practical, as babies with severe pulmonary hypoplasia may undergo FETO. Advantages of this route include the topical application, thus reaching the fetal lung directly, and the low invasiveness of a single procedure that combines FETO and AFSC-EVs. If multiple doses are needed, serial amnioinfusion or fetal intravenous administration could be considered. Serial amnioinfusion is a therapy that has been tested in fetuses with intrauterine renal failure or severe renal anomalies.^137,138^ A prospective, nonrandomized clinical trial reported that serial amnioinfusion decreased fetal mortality but was associated with preterm delivery.^138^ On the other hand, a recent study reported a case of in-utero enzyme-replacement therapy for infantile-onset Pompe’s disease with multiple infusions through the umbilical vein between 24 and 34 weeks of gestation.^139^ This study demonstrated that repeated intravenous injections are feasible as an alternative access to the fetus. Lastly, there is also a possibility that AFSC-EVs can be injected directly into the mother with bioengineered AFSC-EVs customized to target the fetal lung. As no homing mechanism has been described for EVs, research groups have been working on ways to engineer the EVs by modifying surface ligands and receptors.^140^ Nonetheless, to the best of our knowledge there is no experience on surface modification of AFSC-EVs reported in the literature to date. One must consider the potential side effects of EV therapy not only to the fetus but also to the mother. As EVs can cross the blood-brain and placental barrier, careful pharmacodynamic and pharmacokinetic studies should be conducted to exclude off-target effects.^141,142^

Pharmacokinetically, bioluminescence biodistribution studies of labeled AFSC-EVs in the rat model of CDH revealed uptake of AFSC-EVs to the fetal lung, brain, liver, and kidney when administered intra-amniotically.^24^ When administered intravenously, labeled AFSC-EVs were detected in fetal organs as well, but to a lesser extent. Pharmacodynamically, the experimental effects of AFSC-EVs on fetal lung growth, maturation, vascularization, and inflammation have been thoroughly tested and reported.^16,24,33,45,73-77^ Preliminary results have shown anti-inflammatory effects on fetal rat brains secondary to CDH and further studies are underway to exclude detrimental effects on fetal liver and kidney development, as those organs can be affected by maternal and prenatal drug administration such as steroids.^143,144^

Despite these imminent challenges, the field of EV-based research is moving fast to adopt EVs as therapeutics agents, as proven by numerous studies and ongoing trials (Table 2). Undoubtedly, there are several advantages to using EVs as a therapy for several diseases. This includes EVs as a cell-free therapy that bypasses the challenges of cell-based therapies, such as immunogenicity, potential teratogenicity, and cell reproducibility.^145^ EVs are naturally secreted by all cells and regarded as relatively stable and capable of remaining in the circulatory system for extended periods compared to other synthetic drug delivery systems such as liposomes.^146^ The heterogenous cargo can also be advantageous, especially to treat diseases caused by the imbalance of multiple pathways, such as CDH. Despite decades of research, the pathophysiology of CDH and pulmonary hypoplasia remains elusive, and genetic factors are only responsible in approximately 30% of patients.^22,147^ With a multitude of bioactive molecules within their cargo, EVs may target different dysregulated signaling pathways and overcome the limitations of single-drug therapy.

## Conclusions

Fetal pulmonary hypoplasia is characterized by the disruption of multiple signaling pathways that lead to impaired lung growth, epithelial and mesenchymal maturation, and vascularization. A novel antenatal treatment strategy such as AFSC-EVs holds great regenerative potential as their beneficial effects are attributed to the release of several bioactive molecules including miRNAs that modulate several signaling pathways responsible for lung development. Moreover, CDH fetal lungs have a multilineage inflammatory profile, which is an ideal target for AFSC-EV anti-inflammatory properties. Despite the exciting results in different animal models, several steps must be undertaken before this treatment strategy can be translated into clinical practice for babies with pulmonary hypoplasia. This includes the employment of large animal models, such as the lamb, which allows to investigate of the optimal dose and route of administration, as well as potential side-effects for the fetus and the mother.

## Data Availability

No new data was generated for this concise review article.
